# Evaluating the relationship between *Helicobacter pylori* infection **and carotid intima-media thickness a cross sectional study**

**DOI:** 10.1016/j.amsu.2021.102659

**Published:** 2021-08-17

**Authors:** Hamid Reza Talari, Rezvan Moniri, Mohammadreza Mollaghanbari, Hamed Haddad Kashani, Mohammad Naser Jalalian

**Affiliations:** a Anatomical Sciences Research Center, Institute for Basic Sciences, Kashan University of Medical Sciences, Kashan, Iran; b Department of Radiology, School of Medicine, Kashan University of Medical Sciences, Kashan, Iran; c Department of Internal Medicine, School of Medicine, Kashan University of Medical Sciences, Kashan, Iran

**Keywords:** *Helicobacter pylori*, Carotid intima-media thickness, Cytotoxin associated gene A, Atherosclerosis

## Abstract

**Introduction:**

*Helicobacter pylori* is a gram-negative spiral bacterium that is frequently found in the human stomach. Significant association has been reported between Cytotoxin associated gene A (CagA)- positive *Helicobacter pylori* strains and coronary heart disease. The aim of the present study is to investigate the carotid intima-media thickness as an indicator of atherosclerosis in people with *Helicobacter pylori* infection.

**Methods:**

This study was done on patients who underwent upper GI endoscopy and biopsy, and after obtaining conscious consent underwent ultrasound of the right and left carotid arteries for measuring carotid intima-media thickness (CIMT) and blood tests.

**Results:**

In this study, 90 patients who underwent upper GI endoscopy were examined in three groups: negative H. pylori negative, positive cagA and negative cagA. The right, left and average of CIMT in cagA-positive group were significantly higher than the other two groups (p < 0.05). Howerver, the average of CIMT was not significantly different between men and women. Also, the hsCRP average level in positive cagA group was significantly higher than other groups (p < 0.05).

**Conclusion:**

Our findings suggest that there is an increase in CIMT values in patients with H. pylori infection, especially in cases of positive cagA. The positive cagA group showed significantly higher levels of hs-CRP, as a marker of elevated inflammatory response. Therefore, *H. pylori* infection, especially cagA-positive strains and its associated systemic inflammatory response can be considered as a contributing factor in atherosclerosis and cardiovascular disease.

## Highlights

• *H.pylori* infection especially in case of positive CagA^+^ caused the right/left CIMT increase.
• Higher levels of inflammation in *H.pylori*, CagA patients and atherosclerosis risk factors couldn’t cause the significant difference.
• *H.pylori* infection with positive CagA and its inflammation is an important factor in atherosclerosis and cardiovascular diseases.

## 1 Introduction

*Helicobacter pylori* is a spiral gram-negative bacterium that normally colonizes in human gastric epithelium. At least 50% of the world population is infected with this bacterias, which is much higher in developing countries such as Iran that is estimated at up to 90 percent. Lots of studies showed the strong relation between this infection and chronic gastritis, peptic ulcer and gastric carcinoma [1]. These studies showed the relation between infection and some diseases like metabolic disorders such as diabetes [2], Neurological disorders especially stroke [3], Psychiatric complications [4], Gynecological diseases like Hyperemesis gravidarum [5], Pre-eclampsia [6], Infertility [7], eye deseases Glaucoma [8], skin diseases like Chronic resistant urticaria[9], *alopecia* areata [10] and Behcet's syndrome [11], Ear, nose and throat benign diseases [12], and Malignant deseases like laryngeal carcinoma [13], lung cancer [14], hematologic disorders like iron deficiency anemia [15] and Idiopathic thrombocytopenic purpura [16] Hepatobiliary system disorders [17] and cardiovascular disease.

Patients with *H.pylory* infection show high systemic inflammatory symptoms that are with the increase in number of neutrophils and basophils and cytokines and vasoactives [18]. All of these are involved in pathogenicity of extragastric diseases. Atherosclerosis is a chronic inflammatory diseases of arteries [19]. Some researches have proved the relation between infectious pathogens like chlamydia pneumoniae [20], cytomegalovirus [21] and helicobacter pylori and athrosclrosis in coronary and carotid artery [22]. Virulence of pathogens can be an important indicator of their potential for damage and atherosclerosis. The most invasive strains of Helicobacter pylori produce high molecular weight toxins which called Vacuolating cytotoxin A (VacA) and cause the vacuolization of gastricepithelia cells. This toxin can damage the gastric epithelial cells and lead to a local inflammation response. There is an immunogenic protein with VacA which called Cytotoxin associated gene A (CagA). Positive CagA serologically is a common method for *H.pylori* infection diagnosis [23]. Zhang L et al., in 2019 had a study in which 13,168 patients went under carotid ultrasonic examination and urea breath test for *H.pylori*. Examinations showed that *H.pylori* increased the risk of atherosclerosis in men [24]. Dong, X et al., in 2018 showed that carotid intima thickness in patients with positive *H.pylori* was higher than negative *H.pylori* patients. They concluded that *H.pylori* infection had a correlation with carotid intima thickness and caused the carotid artery thickness [25].

Since the exact relation between atherosclerosis and *H.pylori* infection was not clear, in this study carotid intima-media thickness (CIMT) as an atherosclerosis indicator was assessed in patients with *H.pylori* infection.

## 2 Material and methods

**Fig. 1:**
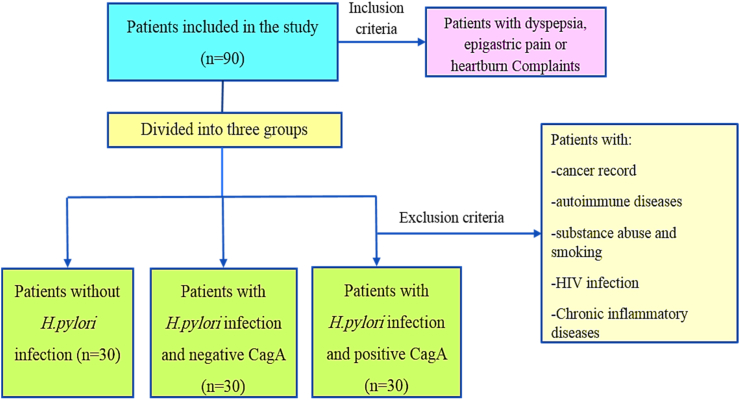
Flowchart of patients who entered the cross sectional study.

90 patients who underwent the endoscopy in 2018–2019 were included in the cross sectional study. Demographic and clinical variables like age, gender, and BMI, cardiovascular disease family record, blood pressure, diabetes, smoking, and peptic ulcer record were evaluated[26] (Fig. 1).

Atherosclerosis risk factors like total cholesterol, low-density lipoprotein (LDL), high-density lipoprotein (HDL), triglyceride (TG), fasting blood sugar (FBS), and high sensitive C - reactive protein (hsCRP) were assessed in blood samples of patients. Gastroduodenoscopy was done for all patients in order to detect the *H.pylori* infection and the sample was taken from the gasteric antrum. Hematoxylin and Eosin (H&E) and Giemsa staining and also urea fast test were done in this sample. If these tests were negative, Helicobacter pylori infection was considered negative. In addition, *H.pylori* culture was done for positive H.pylori patients. Firstly Samples were cultured in a proper culture medium. Then *H.pylori* DNA was extracted and the CagA gene was detected by the PCR method. On this basis, patients were divided into three groups on the basis of positive/negative *H.pylori* and CagA. CIMT was evaluated by ultrasonography method for all patients. Measured IMT in the common left/right carotid artery was recorded as the final CIMT. CIMT and hsCRP as the general inflammation markers were compared in *H.pylori* patients with positive and negative CagA and control groups.

### 2.1 Statistical analysis

Data were analyzed by SPSS version 20.0 software. For assessing of normal distribution, Kolmogorov–Smirnov test was done. Parameteric test like ANOVA and independent *t*-test was used for significance assess. P-value <0.05 was considered significant.

### 2.2 Ethical considerations

The study was approved by the ethics committee of Kashan University of medical science, with ID IR.KAUMS.MEDNT.REC.1397.50. Patients were sure that their information was private and there was no interruption or additional cost in their treatment.

## 3 Results

This study was done in order to evaluate the relation between *H.pylori* infection and carotid intima-media thickness (CIMT). 65.6% of patients were women and 34.4% were men. The average age of patients was 46.10 ± 15.236. The average age of women was 47.22 ± 14.835 which is higher than the other group. But according to the independent *t*-test, there is no significant difference between two groups.

The most common reason for endoscopy was dyspepsia (67.8%) and after that people with abdominal pains and heart burn were frequent.

The average of fasting blood sugar was 93.00 ± 8.41 mg/dl, triglyceride was 148.02 ± 5543 mg/dl, total cholesterol was 191 ± 40.93 mg/dl, LDL was 115.30 ± 25.16 mg/dl, HDL was 46.37 ± 12.89 mg/dl, systolic blood pressure was 118.72 ± 17.26 mmHg, diastolic blood pressure 73.89 ± 12.40 mmHg, BMI 25.18 ± 3.63 kg/m^2^ and hsCRP was 4.27 ± 3.05 mg/l. The average of right CIMT in patients with *H.pylori* was 0.729 ± 0.202 mm and according to the ANOVA test, it was significantly higher than the patients without *H.pylori* infection.The average of left CIMT in patients with *H.pylori* was 0.718 ± 0.183 mm and according to the ANOVA test, it was significantly higher than the other group. The average of both CIMT in patients with *H.pylori* infection was 0.723 ± 0.190 mm and significantly was higher than the other group (p < 0.05) (Table 1).

**Table 1:** The average of CIMT in terms of *H.pylori* infection.

Artery	*H.pylori*	Number	Minimum	Maximum	Average	Standard deviation	aP-value
Right carotid	positive	60	0.36	1.15	0.729	0.202	0.000
	negative	30	0.31	0.79	0.549	0.168	
Left carotid	positive	60	0.37	1.10	0.718	0.183	0.000
	negative	30	0.34	0.93	0.559	0.162	
Both carotid	positive	60	0.365	1.12	0.723	0.190	0.000
	negative	30	0.325	0.86	0.554	0.160	

a P-value <0.05 was considered significant.

In *H.pylori* patients with positive cagA, the average of right CIMT was 0.85 ± 0.142 mm and according to the independent *t*-test was significantly higher than the other group. The average of left CIMT in *H.pylori* patients with positive cagA was 0.825 ± 0.127 mm. The average of both CIMT in patients with positive cagA was 0.837 ± 0.130 mm and independent *t*-test showed that it was significant (p < 0.05) (Table 2).

**Table 2:** The comparison of CIMT average in terms of CagA.

Artery	CagA	number	minimum	maximum	average	Standard deviation	Independent *t*-test
							ap-value
Right carotid	positive	30	0.42	1.15	0.850	0.142	0.032
	negative	30	0.36	0.98	0.609	0.182	
Left carotid	positive	30	0.44	1.10	0.825	0.127	0.021
	negative	30	0.37	0.95	0.611	0.169	
Both carotid	positive	30	0.43	1.12	0.837	0.130	0.023
	negative	30	0.365	0.96	0.610	0.172	

a P-value <0.05 was considered significant.

According to the ANOVA test, the average of right/left/both CIMT in *H.pylori* patients with positive cagA were significantly higher than the negative cagA patients and patients without *H.pylori* infection (Table 3) (see Table 4).

**Table 3:** The average of CIMT in patient with *H.pylori* and positive/negative cagA and without *H.pylory*.

Artery	CagA	Number	Minimum	Maximum	Average	Standard deviation	aP-value
Left carotid	Non-infected	30	0.34	0.93	0.559	0.162	0.000
	Positive cagA	30	0.44	1.10	0.825	0.127	
	Negative cagA	30	0.37	0.95	0.611	0.169	
Both arteries	Non-infected	30	0.325	0.86	0.554	0.160	0.000
	Positive cagA	30	0.43	1.12	0.837	0.13	
	Negative cagA	30	0.365	0.96	0.610	0.172	

a P-value <0.05 was considered significant.

**Table 4:** Spearman correlation of variables.

Variables	Right CIMT	Left CIMT	Both CIMT	*H.pylori* infection	cagA	hsCRP
Age	0.425^b^	0.520^b^	0.474^b^	−0.035	0.155	0.062
gender	−0.016^b^	0.038	0.09	0.057	0.132	−0.038
BMI	0.270^a^	0.281^b^	0.275^b^	0.148	0.155	0.115
diabetes	0.007	−0.045	−0.032	−0.155	−0.089	−0.153
Cardiovascular diseases	0.175	0.215^a^	0.208^a^	0.120	0.069	0.013
High blood pressure	0.116	0.164	0.128	−0.204	−0.034	−0.118
hyperlipidemi	0.127	0.129	0.110	−0.055	0.000	0.090
Triglycirid level	0.116	0.080	0.100	0.191	0.264^a^	0.069
Total cholestrol	0.133	0.178	0.150	−0.050	0.128	0.106
LDL	0.122	0.215^b^	0.161	−0.018	0.105	0.121
HDL	0.188	0.232^b^	0.201	0.026	0.019	0.003
Fasting blood sugar	−0.056	−0.006	−0.037	−0.053	−0.078	0.043
*H.pylori* infection	0.615^b^	0.559^b^	0.597^b^	1.000	0.500^b^	0.539^b^
cagA	0.638^b^	0.592^b^	0.624^b^	0.866^b^	1.000	0.518^b^
hsCRP	0.439^b^	0.392^b^	0.412^b^	0.610^b^	0.518^b^	1.000

a p-value<0.05was considered significant.

b *p-value*<0.01 was considered significant.

Right CIMT in women was higher than men and the average was 0.674 ± 0.210 mm. But this difference, according to the independent *t*-test, was not significant. Left CIMT was higher in men and the average was 0.699 ± 0.201 mm. This difference was not significant. The average of both CIMT was higher in women (0.668 ± 0.195) but it wasn't significant. The average of hsCRP in patients with *H.pylori* infection and positive cagA was 6.85 ± 3.26 mg/l, in patients with *H.pylori* and negative cagA was 3.95 ± 1.97 ml/l and in patients without *H.pylori* infection was 2.02 ± 1.37. According to the ANOVA test, this difference was significant (p < 0.05).

In terms of spearman correlation, there was a significant relation between age and both side CIMT and the average of both CIMT, between the BMI and both side CIMT and the average of both CIMT, between cardiovascular diseases and lef CIMT and the average of both side CIMT, between triglyceride level and cagS, between total cholesterol and *H.pylori* infection, between LDL and left CIMT, between HDL and left CIMT, between *H.pylori* infection and both side CIMT and the average of both side CIMT, between *H.pylori* infection and cagA, between cagA and both side CIMT and the average of both side CIMT, between cag and *H.pylori* infection, between hsCRP level and cagA, between hsCRP and *H.pylori* infection (Tale 4).

The average of age (49.97 ± 14.35 years old), triglycirid level (166.27 ± 55.43 mg/dl), HDL (47.867 ± 13.61 mg/dl), LDL (119.49 ± 23.95 mg dl), BMI (25.70 ± 3.77 kg/m^2^) in patients with *H.pylori* and positive cagA and the average of FBS (94.167 ± 9.37 mg/dl) in *H.pylori* and negative cagA patients were higher but according to the ANOVA test, they weren't significant.

The average of total cholesterol (205.27 ± 44.47 mg/dl), sistolic blood pressure (127.5 ± 16.44 mmHg) and diastolic blood pressure (77.33 ± 12.71 mmHg) were significantly higher in patients without *H.pylori* infection (p < 0.05) (Table 5).

**Table 5:** The average of variables in patients with *H.pylori* and positive/negative cagA and without *H.pylori*.

Variables	Non-infected	Positive cagA	Negative cagA	aP-value
Age	46.97 ± 17.20	49.97 ± 14.35	41.37 ± 13.08	0.592
Triglycirid level	140 ± 45.8	166.27 ± 55.43	137.8 ± 61.15	0.085
Total cholesterol	205.27 ± 44.47	198.87 ± 40.76	170.4 ± 28.1	0.002
HDL	45.34 ± 13.06	47.867 ± 13.61	45.91 ± 12.28	0.734
LDL	119.7 ± 25.29	119.49 ± 23.95	106.72 ± 24.79	0.071
FBS	93.46 ± 9.25	91.38 ± 6.27	94.167 ± 9.37	0.416
MBI	24.87 ± 3.76	25.70 ± 3.77	24.97 ± 3.41	0.629
Sistolic blood pressure	127.5 ± 16.44	118.5 ± 16.14	110.17 ± 15.11	0.000
Diastolic blood pressure	77.33 ± 12.71	76 ± 11.25	68.33 ± 11.62	0.009

a P-value <0.05 was considered significant.

## 4 Discussion

The current study evaluated the relation between *H.pylori* infection and CIMT in 90 patients who underwent the endoscopy. The results showed that right/lft/both sides CIMTin people with *H.pylori* infection was significantly higher than the other groups. Right/left/both sides CIMT in people with *H.pylori* infection and positive CagA was significantly higher than the other groups. Right/both sides CIMT in people with *H.pylori* infection and positive cagA was significantly higher than without-infection group. Left CIMT in these people was higher but this difference was not significant. Right/left/both sides CIMT in people with infection and negative cagA was higher than people without infection but it wasn't significant. Right/both sides CIMT was higher in women and left CIMT was higher in men. But this difference was not significant. The average of hsCRP in *H.pylori* and positive cagA was significantly higher. Atherosclerosis risk factors such as high blood pressure, diabetes and cholesterol level had no disturbing effects on the results.

Wu, Y., et al., in 2013 had a study on the effect of YKL-40 Overexpression on plaque instability in carotid atherosclerosis with CagA-positive Helicobacter pylori infection. They proved that in H.pylori and positive cagA patients, YKL-40 overexpression resulted to more severe athrosclorosis clinical symptoms [27]. In terms of the relation between athrosclorosis risks and *H.pylori* infection, these findings were related to the current study but YKL-40 expression was not evaluated in this study.

In a study which was done by Longo-Mbenza, B., et al., in 2012, it was indicated that there were relation between cardiovascular diseases risk factors and dcarotid plaque and stroke and *H.pylori* infection. Also there were significant relation between the infection severity in males and cardiovascular diseases [28]. These findings don't agree with this study in terms of the relation between the infection and gender. Park, M.J., et al., in 2011 in a study showed that patients with *H.pylori* infection were at the higher risk of coroner arteries atherosclerosis due to the common cardiovascular risk factors [29]. This study indicated the same results. Niccoli, G., et al., in 2010 proved that anti-CagA antibody in patients with Coronary artery disease were higher than the patients with normal coronary arteries. They also indicated that there was a significant correlation between anti-cagA antibody levels and athrosclorosis levels. In addition, positive cagA patients had higher levels of coronary artery disease than negative cagA patients. Therefore they suggested there is significant relation between *H.pylori* infection with positive cagA and athrosclorosis risks [30]. The current study also indicated this relation. Franceschi, F., et al., in 2009 suggested that an intense immune response to cagA-positive *H.pylori* infection might be important for coronary instability mediated by antigen mimicry between CagA antigen and a protein contained in coronary atherosclerotic plaques [31]. The present study is consistent with the significant association between the increased risk of atherosclerosis and CagA-positive *H.pylori*, as well as the association between CagA-positive and high immune response measured here by hsCRP.

## 5 Conclusion

It was concluded that *H.pylori* infection especially in case of positive CagA caused the right/left CIMT increase but the difference between two genders was not significant. Also there were higher levels of inflammation in *H.pylori* and positive CagA patients and atherosclerosis risk factors as confusing factors couldn't cause the significant difference. So it was concluded that *H.pylori* infection with positive CagA and its inflammation is an important factor in atherosclerosis and cardiovascular diseases.

## Funding

The financial support for the current research was provided by Research Deputy of 10.13039/501100004048Kashan University of Medical Sciences, Kashan, Iran.

## Availability of data and materials

The dataset used in this study is available with the authors and can be made available upon request.

## Authors’ contributions

All the authors participated in the study design. HRT, RM, MRM and MNJ collected and documented the data and assisted in preliminary data analysis. HHK and MNJ wrote the initial draft. HRT and HHK participated in draft revision, data analysis and editing of the final draft.

## Consent for publication

Not applicable.

## Ethics approval and consent to participate

All procedures performed in studies involving human participants were in accordance with the ethical standards of the institutional and national research committee and with the 1964 Helsinki declaration and its later amendments.

## Provenance and peer review

Not commissioned, externally peer-reviewed.

## Declaration of competing interest

The authors declared that they have no competing interests.

## Abbreviations

CIMT: Carotid intima-media thickness
IMT: Intima-media thickness
*H.pylori*: *Helicobacter pylori*
LDL: Low-density lipoprotein
HDL: High-density lipoprotein
TG: Triglyceride
FBS: Fasting blood sugar
hsCRP: High sensitive C - reactive protein
CagA: Cytotoxin associated gene A
